# Thy-AuNP-AgNP Hybrid Systems for Colorimetric Determination of Copper (II) Ions Using UV-Vis Spectroscopy and Smartphone-Based Detection

**DOI:** 10.3390/nano12091449

**Published:** 2022-04-24

**Authors:** Thitiporn Thongkam, Amara Apilux, Thitaporn Tusai, Tewarak Parnklang, Sumana Kladsomboon

**Affiliations:** 1 Department of Clinical Chemistry, Faculty of Medical Technology, Mahidol University, Phutthamonthon, Nakhon Pathom 73170, Thailand; thipor.tin@gmail.com (T.T.); amara.apl@mahidol.edu (A.A.); 2 Community Health Care Service, Faculty of Medical Technology, Mahidol University, Wang Lang Road Siriraj, Bangkok 10700, Thailand; thitaporn.tus@mahidol.ac.th; 3 Research Network NANOTEC-CU on Advanced Structural and Functional Nanomaterials, Chulalongkorn University, Bangkok 10330, Thailand; tewarak.p@sci.kmutnb.ac.th; 4 Department of Radiological Technology, Faculty of Medical Technology, Mahidol University, Phutthamonthon, Nakhon Pathom 73170, Thailand

**Keywords:** copper (II) ion detection, gold nanoparticles, silver nanoparticles, thymine, smartphone, colorimetric probe

## Abstract

A colorimetric probe based on a hybrid sensing system of gold nanoparticles (AuNPs), silver nanoparticles (AgNPs), and thymine (Thy) was developed for easy and rapid detection of copper (II) ions (Cu^2+^) in solution. The underlying principle of this probe was the Cu^2+^-triggered aggregation of the nanoparticle components. Color change of the sensing solution (from red to purple) was clearly observed with naked eyes. The experimental parameters, including pH and concentration of tris buffer, thymine concentration and AgNP dilution ratios, were investigated and optimized. Once optimized, the limits of detection were found to be 1, 0.09 and 0.03 ppm for naked eyes, smartphone application and UV-vis spectrophotometer, respectively. Furthermore, determination of Cu^2+^ was accomplished within 15 min under ambient conditions. For quantitative analysis, the linearity of detection was observed through ranges of 0.09–0.5 and 0.03–0.5 ppm using smartphone application and UV-vis spectrophotometer, respectively, conforming to the World Health Organization guideline for detection of copper at concentrations < 2 ppm in water. This developed hybrid colorimetric probe exhibited preferential selectivity toward Cu^2+^, even when assessed in the presence of other metal ions (Al^3+^, Ca^2+^, Pb^2+^, Mn^2+^, Mg^2+^, Zn^2+^, Fe^3+^, Ni^2+^, Co^2+^, Hg^2+^ and Cd^2+^). The developed procedure was also successfully applied to quantification of Cu^2+^ in real water samples. The recovery and relative standard deviation (RSD) values from real water sample analysis were in the ranges of 70.14–103.59 and 3.21–17.63%, respectively. Our findings demonstrated a successful development and implementation of the Thy-AuNP-AgNP hybrid sensing system for rapid, simple and portable Cu^2+^ detection in water samples using a spectrophotometer or a smartphone-based device.

## 1. Introduction

Copper (Cu) is an essential trace element which the human body requires for maintaining several biological processes, including cellular respiration, iron transport, enzyme function and brain development [1,2,3]. Nevertheless, excess amounts of Cu can adversely affect human health, causing vomiting, abdominal pain, decreased liver function and even neurological impairment [4,5]. To protect against these undesirable effects, the World Health Organization (WHO) recommends that the Cu concentration in water not exceed 2 ppm (or mg/L) [6]. Cu can be found in both natural and anthropogenic sources [7]. A major source of Cu release into the environment is from industrial processes [1], including the fertilizer, mining, battery, electronics and machinery industries [8]. Therefore, the presence of Cu-contaminated water is frequently reported in industrial development zones [9,10,11]. In Thailand, Cu contamination was consistently found in industrial estate areas, including the Wang Saphung district in Loei province, Map Ta Phut industrial estate in Rayong province, Mueang Samut Sakhon district in Samut Sakhon province, Tha Tum district in Prachinburi province [10] and Bangpa-In district in Phra Nakhon Si Ayutthaya province [12,13]. The release of Cu into the environment, especially surface waters [14], may harm plants, fish and invertebrates and be transferred into humans via the food chain [3,15]. Routine monitoring of Cu contamination for health risk assessment is highly recommended to ensure that Cu levels are not exceeding standard levels. Therefore, a rapid and convenient method of Cu determination in environmental samples is imperatively needed.

Copper generally exists in one of two forms, i.e., insoluble cuprous (Cu^1+^) and soluble cupric (Cu^2+^) forms. Because of their properties, the Cu^2+^ form is the main concern, as a contaminate affecting human health [15]. Several methods for Cu^2+^ detection have been utilized, including inductively-coupled plasma mass spectroscopy (ICP-MS), atomic absorption spectroscopy (AAS), electrochemistry [16,17], fluorescence [18,19] and colorimetry [20,21,22]. Among these methods, nanoparticle-based colorimetry provides the greatest advantages, including straightforward operation, fast detection, reasonable cost, and no requirement for sophisticated instruments [23]. Gold nanoparticles (AuNPs) and silver nanoparticles (AgNPs) are the most widely known nanoparticles due to their unique morphologically-dependent optical and electrical properties [24]. Some nanoparticle-based colorimetric detection systems utilize the alteration of the localized surface plasmon resonance (LSPR) upon aggregation and anti-aggregation of the nanoparticles [25]. For example, polyethylenimine (PEI) and D-penicillamine (DPC)-modified gold nanoparticles [26,27] and dopamine and casein peptide-modified silver nanoparticles [28,29] are employed for Cu^2+^ detection. Several organic compounds are eligible for Cu^2+^ ion binding, such as adenine, cytosine, uracil and thymine. Among these compounds, thymine exhibits high interaction energy and excellent charge transfer according to the experimental results and density functional theory calculations [30,31,32].

A previous detection probe for Cu^2+^ was developed using an AuNP system. However, its limit of detection was insufficient for the recommended guideline level [33]. To achieve the required levels of performance with respect to sensitivity and selectivity, a hybrid system based on AuNPs and AgNPs was designed. In this study, a colorimetric probe for Cu^2+^ detection was developed using a hybrid of nanoparticles (AuNPs and AgNPs) in the presence of thymine. The sensitivity and selectivity of this probe were investigated by naked eyes, smartphone application and UV-vis spectrophotometer

## 2. Materials and Methods

### 2.1. Chemicals

All chemicals were analytical reagent (AR) grade. Citrate-capped AuNPs with an average diameter of 10 nm, citrate-capped AgNPs with an average diameter of 10 nm, thymine, Tris (hydroxymethyl) aminomethane powder, i.e., tris buffer, cadmium chloride hemipentahydrate (CdCl_2_·2.5H_2_O) and magnesium chloride hexahydrate (MgCl_2_·6H_2_O) were purchased from Sigma-Aldrich (St. Louis, MO, USA). Calcium chloride anhydrous (CaCl_2_), copper (II) sulphate pentahydrate (CuSO_4_·5H_2_O), iron (III) chloride hexahydrate (FeCl_3_·6H_2_O), manganese chloride tetrahydrate (MnCl_2_·4H_2_O), lead (II) chloride (PbCl_2_), and zinc chloride (ZnCl_2_) were purchased from Bio Basic (Markham, ON, Canada). Aluminum chloride hexahydrate (AlCl_3_·6H_2_O) and mercuric (II) nitrate monohydrate [Hg(NO_3_)_2_·H_2_O] were purchased from Loba Chemie (Mumbai, India). Cobalt (II) chloride hexahydrate (CoCl_2_·6H_2_O) and nickel (II) sulphate hexahydrate (NiSO_4_·6H_2_O) were purchased from Fisher Scientific (Leics, UK). Aqueous solutions were prepared in ultrapure water obtained from Millipore Milli-Q water purification system (Burlington, MA, USA, 18.2 MΩ∙cm). All metal ion stock solutions were prepared in 0.1 M of tris buffer at pH 7.

### 2.2. Apparatus and Measurements

UV-visible absorption spectra were recorded at room temperature using an Evolution 600 UV-vis spectrophotometer (Thermo Fisher Scientific, Waltham, MA, USA). Morphology and size distribution of the nanoparticles were determined using a JEM-1400 transmission electron microscope (TEM) (JEOL, Peabody, MA, USA) operating at 100 kV. The samples were prepared by placing 50 μL of sample solution on the carbon-coated copper grids and subsequently drying in a desiccator overnight before TEM analysis.

### 2.3. Detection of Cu^2+^

Cu^2+^ reaction mixtures were prepared in 0.1 M tris buffer. Typically, 50 μL of citrate-capped AuNPs, 50 μL of citrate-capped AgNPs and 12.5 μL of 0.5 M thymine were mixed with 25 μL of Cu^2+^ sample and incubated at room temperature for 15 min. The aggregation of hybrid components could be observed by naked eyes as the solution’s color shifted from red to purple. Results were quantified by UV-vis spectrophotometer in the range of 400–800 nm. Each experiment was performed in triplicate. The influence of sensing conditions, including pH and concentrations of tris buffer (pH 3–8 and 0–1 M, respectively), thymine concentration (0.0625–0.5 M), and AgNP dilutions in distilled water (DW; undiluted, 1:2, 1:5 and 1:10) were investigated.

### 2.4. Smartphone Application for Cu^2+^ Detection

The developed hybrid sensing system was integrated with a smartphone-based detection platform to achieve on-site detection as well as convenience, simplicity and interpersonal transferability. After 15 min of incubation, the sensing solution was captured with an iPhone 8 Plus smartphone in the light-controlling box in order to suppress an external light interference. The distance between the sensing solution tube and the smartphone camera was approximately 30 cm. The sensing reaction tubes were linearly aligned at an angle of 180° with the smartphone camera. Intensity values of red-green-blue (RGB) color of the captured solutions at a resolution of 12.2 megapixels were extracted using a freely available color-scanning application, i.e., “Color Picker version 3.1” developed by Achim Heyman, installed on an iPhone 8 Plus smartphone. The RGB value of the color of each solution was recorded in the absence and/or the presence of Cu^2+^ at various concentrations. A calibration curve was constructed by plotting the differences of B/R ratios between the sensing solutions injected with various Cu^2+^ concentrations and a blank solution, as a function of Cu^2+^ concentration.

### 2.5. Real Water Analysis

A real water sample employed in this work was drinking water obtained from local grocery stores. All samples were filtered by Whatman No. 1 filter paper to remove impurities and mixed with 1 M tris buffer at a volume/volume ratio of 1:9 prior to analysis. For the recovery study, Cu^2+^ stock solutions were added to the prepared water samples to achieve the final Cu^2+^ concentrations of 0.2, 0.4 and 1 ppm. Finally, the spiked samples at each concentration were analyzed in triplicate with the developed probe.

## 3. Results and Discussion

### 3.1. Principle for Cu^2+^ Detection

**Figure 1:**
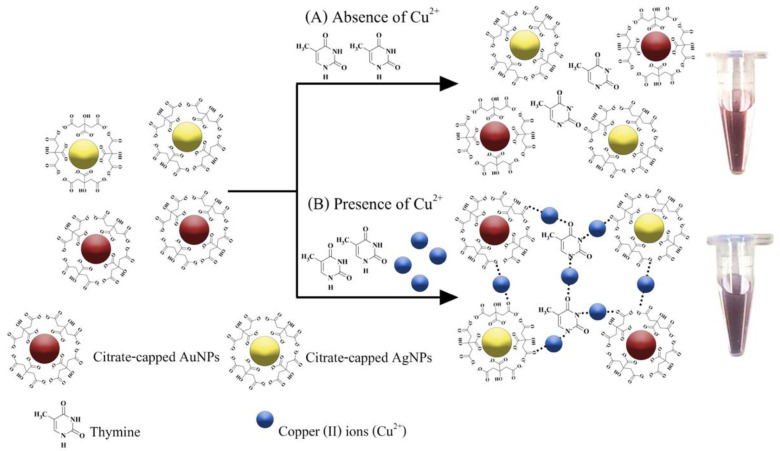
Schematic representation of the principle of colorimetric detection of Cu^2+^ based on a Thy-AuNP-AgNP hybrid system in the (**A**) absence or (**B**) presence of Cu^2+^.

The proposed sensing mechanism of the copper (II) ions (Cu^2+^) detection is shown in Figure 1. Citrate-capped AuNPs and AgNPs, both with average diameters of 10 nm, were used in this detection system. Both nanoparticles possessed carboxyl functional groups on their surface which were deprotonated at pH 7 [34], inducing negatively charged surfaces.

Electrostatic stabilization of the nanoparticles in suspension maintained them in a dispersed state [34,35,36]. In the absence of Cu^2+^ (Figure 1A), the repulsion among AuNPs, AgNPs and thymine (Thy) molecules existed due to the negatively charged nanoparticle surfaces. Consequently, the hybrid system remained in a dispersed state. In contrast, when Cu^2+^ ions were introduced into the suspension (Figure 1B), AuNPs, AgNPs and thymine were congregated through the coordination of Cu^2+^ with oxygen atoms on carbonyl groups and nitrogen atoms on thymine molecules, resulting in aggregation of AuNPs and AgNPs (the aggregated state) [37,38]. Physically, the suspension’s color changed from red to purple, a change which could be clearly observed by naked eyes.

**Figure 2:**
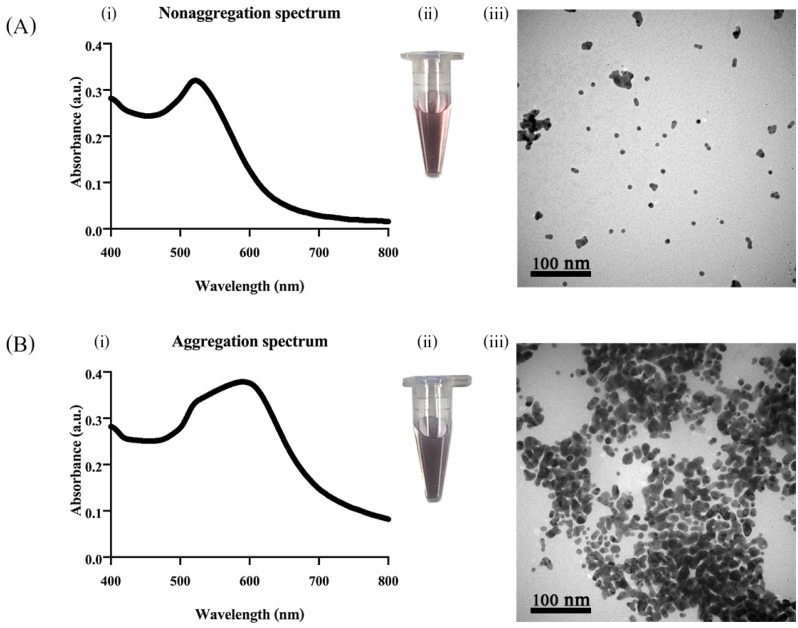
(i) The absorption spectra, (ii) the digital photographs and (iii) the TEM micrographs of the Thy-AuNP-AgNP solutions in the absence (**A**) and the presence (**B**) of Cu^2+^. Scale bars in the TEM micrographs are 100 nm.

To verify the sensing mechanism of the Thy-AuNP-AgNP hybrid system in the presence of Cu^2+^ ions, UV-vis spectroscopy and transmission electron microscopy (TEM) techniques were employed. Figure 2 shows the absorption spectra, TEM images and digital photographs of the Thy-AuNP-AgNP hybrid probe without Cu^2+^ ions and with Cu^2+^ ions. When Cu^2+^ ions were absent, the absorption spectrum exhibited a single absorption peak centered at 520 nm [21,34]. The color of the solution was red (Figure 2A(ii)). Both AuNPs and AgNPs appeared in the dispersed state when observed in TEM micrographs (Figure 2A(iii)). The average diameters of the dispersed AuNPs and AgNPs were essentially the same, i.e., about 10 nm. When Cu ions were present, the LSPR absorption band of the solution at ~520 nm was gradually increased with a new absorption band centered at 600 nm emerging (Figure 2B(i)). The observed LSPR wavelength shift in the absorption spectrum was in agreement with previous reports utilizing AuNPs as sensing materials for colorimetric detection [27,33,39]. With Cu^2+^ ions, the solution’s color shifted from red to purple (Figure 2B(ii)), and the nanoparticles were observed by TEM to be in the aggregated state (Figure 2B(iii)). These results substantiated the Cu^2+^-triggered aggregation of the hybrid Thy-AuNP-AgNP sensing system.

### 3.2. Optimization of Cu^2+^ Sensing Conditions

To obtain optimum conditions for Cu^2+^ detection using the Thy-AuNP-AgNP hybrid system, the possibly influential experimental parameters (pH and concentration of tris buffer, concentration of thymine, and dilution ratio of AgNP) were investigated.

The effectiveness of parameter optimization was quantified as the difference in the absorbance ratio, Δ*A_r_*, defined as follows:

(1) $$\Delta A_{r}={\left[\frac{A_{600}}{A_{520}}\right]}_{sample}-{\left[\frac{A_{600}}{A_{520}}\right]}_{blank}$$

#### 3.2.1. pH of Tris Buffer

**Figure 3:**
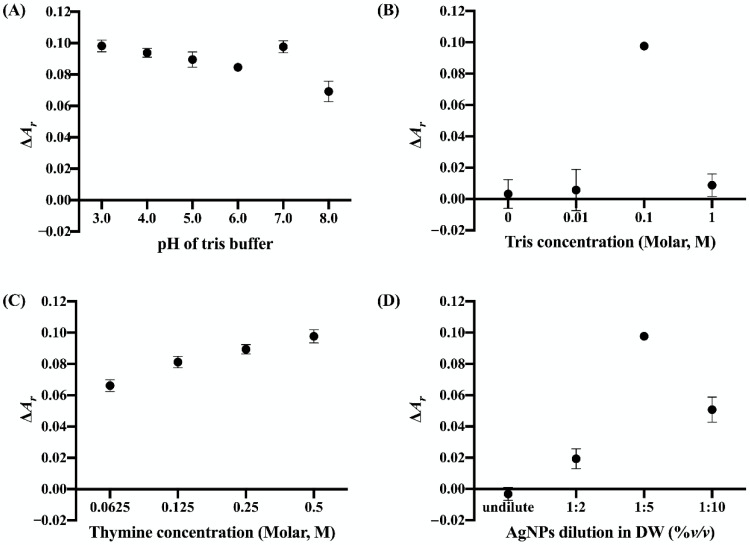
The plots of Δ*A_r_* values against (**A**) pH of tris buffer (pH 3−8), (**B**) concentration of tris buffer (0, 0.01, 0.1, and 1 M), (**C**) concentration of thymine (0.0625, 0.125, 0.25, and 0.5 M), and (**D**) dilution ratio of AgNPs:DW (undilute, 1:2, 1:5, and 1:10).

The pH of sensing systems is a crucial factor affecting the aggregation process of nanoparticles. The pH of the tris buffer was varied from 3 to 8. The blank sample was prepared by mixing AuNPs, AgNPs and 0.5 M thymine with tris buffer (0.1 M) at a specified pH. The test sample was prepared in the same manner of the blank sample, except that 10 ppm of Cu^2+^ ions were injected into the tris buffer solution prior to the final mixing. As shown in Figure 3A, the Δ*A_r_* value reached maxima of 0.098 ± 0.003 at pH 3 and 0.097 ± 0.003 at pH 7. Under acidic conditions (pH < 5.0) [40], citrate-capped nanoparticles are unstable because of the full protonation of passivating citrate molecules, leading to attenuation of negative charges on their surfaces and subsequent aggregation [34]. In addition, the U.S. Environmental Protection Agency recommends that the pH of drinking water should be within the range of 6.5 to 8.5 [41]. Therefore, tris buffer at pH 7 was selected for use in the subsequent experiments.

#### 3.2.2. Tris Buffer Concentration

The concentration of tris buffer is directly associated with the salt effect and its ionic strength. Concentrations of 0, 0.01, 0.1 and 1 M were investigated. The pH of tris buffer was fixed at pH 7. The blank samples were prepared by mixing AuNPs, AgNPs, 0.5 M thymine and tris buffer at the specified concentration (at pH 7). Test samples were prepared in the same manner, except that 10 ppm of Cu^2+^ ions were injected into the tris buffer solution prior to final mixing. As shown in Figure 3B, the Δ*A_r_* values for the tris buffer concentrations of 0, 0.01 and 1 M were not significantly different. At the tris buffer concentrations of 0 and 0.01 M, the Δ*A_r_* values ≈ 0 suggested that there was no aggregation in both blank and test solutions. On the other hand, AuNPs and AgNPs might be aggregated at 1 M tris buffer concentration without addition of Cu^2+^ ions, leading to similar values of [*A*_600_/*A*_520_]_sample_ and [*A*_600_/*A*_520_]_blank_ ≈ 1 and a Δ*A_r_* value ≈ 0. High concentrations of H^+^ from the tris buffer might disrupt the negative charges on the AuNP and AgNP surfaces, leading to the destabilization and aggregation of the nanoparticles in the suspension [42]. The tris buffer concentration at 0.1 M clearly displayed the highest Δ*A_r_* value, implying that the ionic strength might not affect sensitivity of the hybrid Thy-AuNP-AgNP probe. Therefore, 0.1 M tris buffer was chosen for subsequent experiments.

#### 3.2.3. Thymine Concentration

Thymine solution can serve as a recognition unit for Cu^2+^ ion detection based on the molecules’ oxygen and nitrogen atoms [37,38]. The blank sample was prepared by mixing AuNPs, AgNPs and a specified concentration of thymine with 0.1 M tris buffer at pH 7. Test samples were prepared in the same manner as the blank sample, except that 10 ppm of Cu^2+^ ions were injected into the tris buffer solution prior to final mixing. As shown in Figure 3C, 0.5 M thymine generated the highest Δ*A_r_* value. Therefore, 0.5 M thymine was chosen for subsequent experiments.

#### 3.2.4. AgNP Dilution

Citrate-capped AuNPs can be employed as the sole nanoparticle for sensing Cu^2+^ ions. However, the lowest concentration of an analyte that can be detected or limit of detection (LOD ) is reported to be 320 ppm [33], not sufficiently low for practical monitoring of Cu^2+^ in drinking water. In this work, AgNPs were purposefully employed to couple with AuNPs for enhanced Cu^2+^ ion sensitivity. In preliminary studies, considerable difference in [*A*_600_/*A*_520_]_sample_ with the Cu^2+^ concentration of 10 ppm and [*A*_600_/*A*_520_]_blank_ was observed when AgNPs was added to the sensing system comprised of AuNPs, thymine and tris buffer. Therefore, the effect AgNP dilution, i.e., the relative concentration of AgNPs, was evaluated. Stock AgNP suspension was diluted with DW at AgNP:DW volume/volume ratios of 1:2, 1:5 and 1:10. The blank sample was prepared by mixing stock AuNPs, AgNPs at a specified dilution, 0.5 M thymine and 0.1 M tris buffer at pH 7. Test samples were prepared in the same manner, except that 10 ppm of Cu^2+^ ions were injected into the tris buffer solution prior to final mixing. As shown in Figure 3D, the Δ*A_r_* values of the sample with undiluted AgNPs and 1:2 AgNP dilution were not significantly different. However, the values of [*A*_600_/*A*_520_]_sample_ ≈ 1.030 ± 0.006 and [*A*_600_/*A*_520_]_blank_ ≈ 1.023 ± 0.004 with undiluted AgNPs and 1:2 AgNP dilution, respectively, implied LSPR shifts from ~520 to ~600 nm, suggesting aggregation in both the blank and test solutions. The Δ*A_r_* value of the 1:5 AgNP dilution was highest (0.10) of those tested. Therefore, an AgNP dilution of 1:5 was selected for further experiments.

### 3.3. Analysis of Cu^2+^

The Thy-AuNP-AgNP hybrid sensing system under optimal conditions was evaluated for quantitation of Cu^2+^ concentration based on its aggregation effect. A blank solution was prepared by mixing stocks of AuNPs and AgNPs at a 1:5 ratio, 0.5 M thymine and 0.1 M tris buffer at pH 7. Sample solutions were prepared by individually injecting specified Cu^2+^ concentrations (0, 0.01, 0.05, 0.1, 0.25, 0.5, 1, 2.5, 5, 7.5 or 10 ppm) into the blank solution of the Thy-AuNP-AgNP colorimetric probe. Subsequently, the color of the solutions was monitored by both naked eyes and smartphone, and the corresponding absorption spectra were recorded by a UV-Vis spectrophotometer.

#### 3.3.1. Naked Eye Detection and Spectrophotometric Quantification

**Figure 4:**
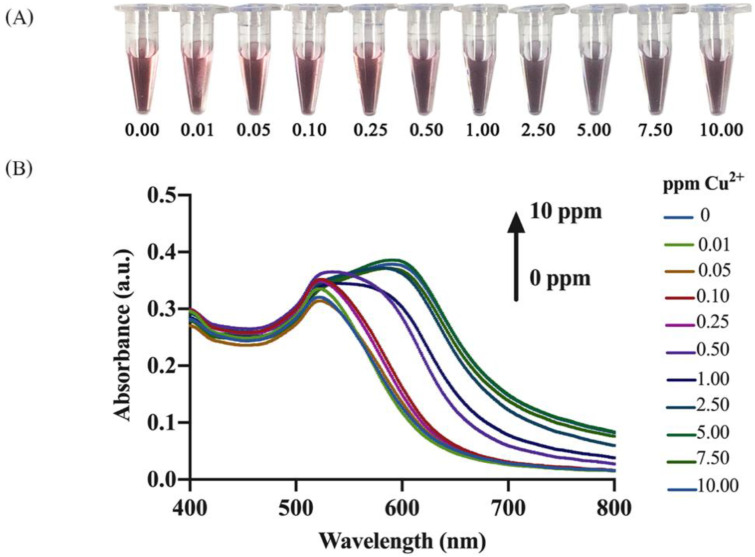
(**A**) Photographs of the Thy-AuNP-AgNP sensing solutions with various Cu^2+^ concentrations (0.00, 0.01, 0.05, 0.10, 0.25, 0.50, 1.00, 2.50, 5.00, 7.50 and 10.00 ppm) and (**B**) the corresponding absorption spectra.

A red-to-purple color shift of the hybrid system appeared in the spiked solutions and was assessed after 15 min of incubation as shown in Figure 4A.

**Figure 5:**
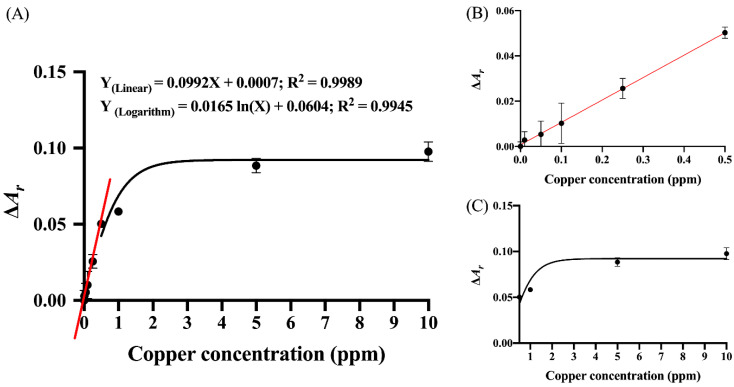
(**A**) The plot of Δ*A_r_* against the Cu^2+^ concentration in the range of 0–10 ppm. Each experiment was performed in triplicate. The linear regression line for Cu^2+^ concentration in the range of 0.03–0.5 ppm is indicated with a red line. The logarithmic regression line for Cu^2+^ concentration in the range of 0.5–10 ppm is indicated with a black line. Insets (**B**,**C**) are the expanded view of the linear and logarithmic regression lines, respectively. The linear and logarithmic equations along with the corresponding correlation coefficients are indicated in (**A**).

The color shift could be discriminated by naked eyes when >1.0 ppm of Cu^2+^ ions were injected into the hybrid sensing system. These observations were corroborated by the shifts of absorption peak observed in the corresponding absorption spectra (Figure 4B). When the concentration of Cu^2+^ increased, the absorption peak intensity at ~520 nm showed a slight increase compared to a considerable increase of the absorption peak intensity at ~600 nm. The quantitative determination of Cu^2+^ concentration was performed through the Δ*A_r_* value. A calibration curve was constructed by plotting Δ*A_r_* values as a function of Cu^2+^ concentration (Figure 5). Statistical analysis of the calibration plot revealed in a linear relationship between Δ*A_r_* and Cu^2+^ concentration in the range of 0.03–0.05 ppm, while a logarithmic relationship was seen with concentrations in the range of 0.5–10 ppm. The linear and logarithmic equations were Y(Linear) = 0.0992X + 0.0007 and Y(Logarithm) = 0.0165ln(X) + 0.0604, respectively, where Y is Δ*A_r_* and X is Cu^2+^ concentration. The correlation coefficients (R^2^) for the linear and the logarithmic regression lines were 0.9989 and 0.9945, respectively. The LOD was calculated by 3σ/slope, where the slope was derived from the corresponding linear calibration curves of the analytes and σ was the standard deviation (SD) from the corresponding linear regression lines [43]. The LOD of the developed probe was 0.03 ppm. Table 1 compares the analytical performances in terms of the LODs and analysis time of the developed hybrid sensing system along with previously reported colorimetric probes for speed and limit of detection of Cu^2+^ based on gold and silver nanoparticles. The LOD of 0.03 ppm of Cu^2+^ from our developed probe was sufficiently low to be useful in monitoring drinking water according to the WHO guideline (<2 ppm) [44].

#### 3.3.2. Smartphone-Based Detection

To endow the developed Thy-AuNP-AgNP hybrid sensing system with convenience, portability, rapidity, on-site analysis, real-time quantification and cost-effective operation, the developed hybrid system was integrated with a smartphone platform. Nowadays, smartphones are powerful mobile devices which offer built-in cutting-edge cameras, operating software and hardware technologies, color displays and communication functionality [48]. Despite its sophisticated capabilities, a person new to a smartphone can become familiarized with it in no time [49]. Therefore, it might accelerate the speed to peak performance of a new analyzer performing this complex chemical analysis [50]. In this work, it was employed for colorimetric analysis through a smartphone-based application, i.e., “Color Picker”. This application monitored the change of RGB color of hybrid sensing solutions which resulted from the presence of Cu^2+^. The RGB values were recorded by a whole number from 0 to 255 in the form of [255,255,255], where each number represents the red, green and blue color, respectively.

As the hybrid sensing system shifted from red to purple, the B/R ratio was used as the representative variable for quantification of Cu^2+^. Efficiency of the smartphone integration was assessed by the difference in the *B/R* ratio, Δ(*B/R)_r_*, defined as follows:

(2) $$\;\Delta {\left[\frac{B}{R}\right]}_{r}={\left[\frac{B}{R}\right]}_{sample}-{\left[\frac{B}{R}\right]}_{blank}$$

**Figure 6:**
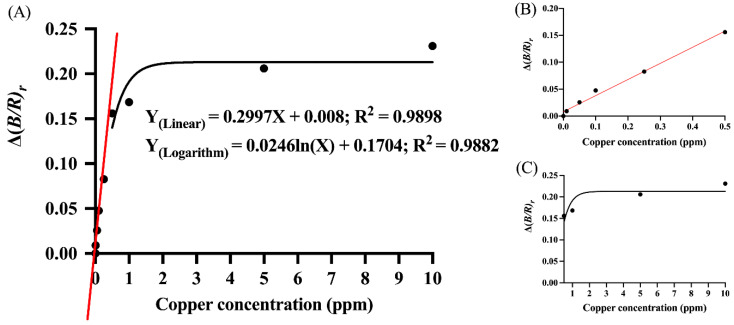
(**A**) The plot of Δ(*B/R)_r_* against Cu^2+^ concentrations in the range of 0–10 ppm. The linear regression line for Cu^2+^ concentrations in the range of 0.09–0.5 ppm is indicated with a red line. The logarithmic regression line for Cu^2+^ concentrations in the range of 0.5–10 ppm is indicated with a black line. Insets (**B**,**C**) are expanded views of the linear and logarithmic regression lines, respectively. The linear and logarithmic equations along with the corresponding correlation coefficients are indicated in (**A**).

As shown in Figure 6, the linear calibration plot of Δ(*B/R)_r_* against Cu^2+^ concentration was determined for Cu^2+^ concentrations in the range of 0.09–0.5 ppm. The linear equation and its corresponding correlation coefficient was Y(Linear) = 0.2997X + 0.008 and R^2^ = 0.9898, respectively, where Y is Δ(*B*/*R)_r_* and X is Cu^2+^ concentration. The logarithmic regression line, Y(Logarithm) = 0.0246ln(X) + 0.1704, with the corresponding correlation coefficient of 0.9882 was obtained for Cu^2+^ concentrations in the range of 0.5–10 ppm. Both linear and logarithmic fittings using data from the smartphone-based platform exhibited trends identical to those of spectrophotometric-based analysis (Figure 5). The LOD for the RGB application was 0.09 ppm.

### 3.4. Selectivity Test

**Figure 7:**
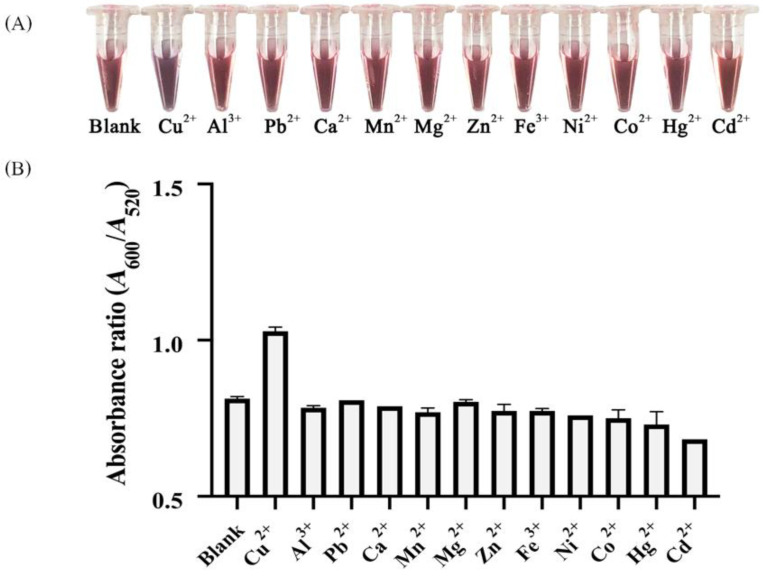
(**A**) Color changes and (**B**) absorption peak ratios at 600 nm and 520 nm, i.e., *A*_600_ /*A*_520_, of the hybrid sensing solutions in the presence of 1 ppm of the tested metal ions.

To evaluate the selectivity of the developed probe for Cu^2+^ detection, a test was performed with other environmental metals (Al^3+^, Ca^2+^, Pb^2+^, Mn^2+^, Mg^2+^, Zn^2+^, Fe^3+^, Ni^2+^, Co^2+^, Hg^2+^ and Cd^2+^). The permissible limits of metal concentrations in drinking water are recommended by a diverse group of authorities, including APHA, WHO, ISI, CPCB and ICMR. For most heavy metals, concentrations considered safe in drinking water range up to 0.003–3 ppm [51,52]. Based on those recommended limits, we selected the concentration of 1 ppm for evaluation of our probe’s selectivity. Among the many environmental ions tested, only Cu^2+^ ions brought about a red-to-purple color shift in the hybrid sensing solution. Notable color change was not observed in the presence of Al^3+^, Ca^2+^, Pb^2+^, Mn^2+^, Mg^2+^, Zn^2+^, Fe^3+^, Ni^2+^, Co^2+^ nor Cd^2+^ (Figure 7A). Furthermore, the absorbance responses of these metal ions tested for interference were nearly identical with those of the blank solution (Figure 7B) and their differences were negligible.

**Figure 8:**
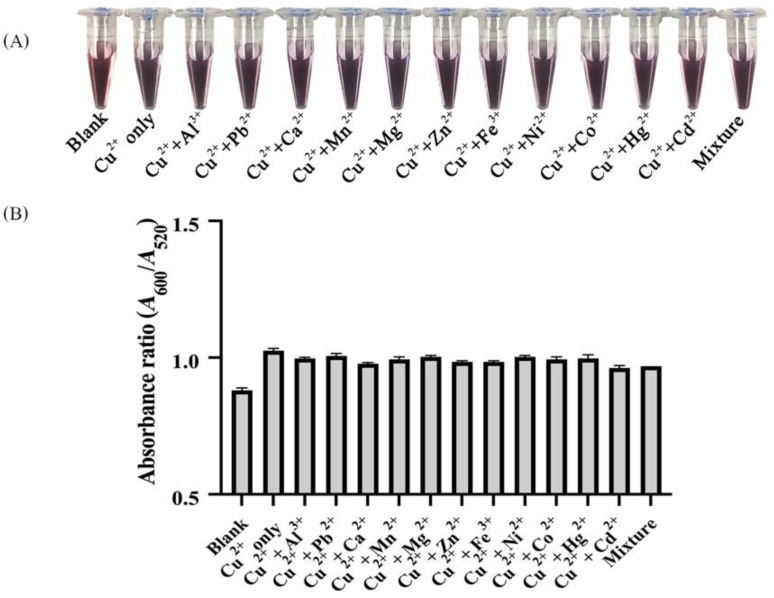
(**A**) Color changes and (**B**) the absorption peak ratios at 600 nm and 520 nm, i.e., *A*_600_ /*A*_520_, of solutions containing 1 ppm Cu^2+^ ion and 1 ppm of the indicated interference metal ions, as well as a solution mixing 1 ppm of these eleven interference metal ions (mixture).

The presence of other metal ions exhibited very little interference with the detection of Cu^2+^. It was observed that Al^3+^, Ca^2+^, Pb^2+^, Mn^2+^, Mg^2+^, Zn^2+^, Fe^3+^, Ni^2+^, Co^2+^, Hg^2+^ and Cd^2+^ did not significantly interfere with Cu (II) detection by the hybrid Thy-AuNP-AgNP system (Figure 8). In addition, the solution containing eleven potentially interfering metals (Al^3+^, Ca^2+^, Pb^2+^, Mn^2+^, Mg^2+^, Zn^2+^, Fe^3+^, Ni^2+^, Co^2+^, Hg^2+^ and Cd^2+^) was considered. The color of the multi-ion solution was similar to the color of the Cu^2+^ only solution. Thus, none of these metal ions, alone or combined, significantly affected this detection system. These results suggested that the developed probe was appropriate for selective determination of Cu^2+^ in aqueous solution.

### 3.5. Real Sample Analysis

To demonstrate the practical application of the Thy-AuNP-AgNP hybrid sensing system, it was applied to determine Cu^2+^ concentration in samples of local drinking water. The real water samples were spiked with Cu^2+^ to give concentrations of 0.2, 0.4 and 1 ppm. Subsequently, the detected values, the relative standard deviations and the recovery values were determined. The results are summarized in Table 2. The samples were also analyzed by atomic absorption spectrometer (AAS) to confirm the Cu^2+^ concentrations. The average concentration by AAS of unspiked water samples was 0.02 ppm, which was lower than the LOD of the developed probe. The detected concentrations of spiked samples obtained by UV-vis spectrophotometer were found to be 0.140, 0.336 and 0.835 ppm for the real water samples with final concentration of 0.2, 0.4, and 1.0 ppm Cu^2+^, respectively. When the smartphone-based RGB application was employed, the detected concentrations were 0.203, 0.361, and 1.036 ppm for these same spiked samples, respectively. The average values of recovery and relative standard deviation (RSD) from the spectrophotometric measurement were 70.14–84.01% and 3.21–17.63%, respectively. The average recovery and the average RSD values were in the ranges of 90.32–103.59% and 6.26–9.49%, respectively, for the smartphone-based system. These results were acceptable in accordance with the Codex Alimentarius Commission guidelines, which state that acceptable mean recoveries (for enforcement purposes) should normally be within a range of 70–120%, with an RSD ≤ 20% [53]. Therefore, the developed probe reliably quantified Cu^2+^ concentration in real water samples.

## 4. Conclusions

A colorimetric probe based on a Thy-AuNP-AgNP hybrid system was successfully developed for simple and rapid copper (II) ions (Cu^2+^) detection. The sensing mechanism was based on Cu^2+^-induced aggregation of the hybrid system. The color change of the sensing solution from red to purple was triggered by introducing Cu^2+^ to the hybrid system and could be detected by naked eyes. The developed probe might be applied for Cu^2+^ quantification on various platforms, including conventional UV-visible spectrophotometer, portable smartphone devices and naked eyes, with LODs of 0.03, 0.09 and 1 ppm, respectively. These LODs were less than those recommended in the WHO guideline for copper concentration in water. The developed probe successfully quantitated Cu^2+^ in real water samples. The recovery and the relative standard deviation (RSD) values from spiked real water samples were in the ranges of 70.14–103.59 and 3.21–17.63%, respectively. These findings demonstrated that the developed probe for Cu^2+^ determination featured simplicity, cost-effectiveness, rapid detection, portability and prevention of misinterpretation by untrained persons. The proposed probe has much promise for on-site Cu^2+^ monitoring of environmental samples.

## Figures and Tables

**Table 1:** Comparison of colorimetric detection probes for Cu^2+^ based on AuNPs and AgNPs, and this Thy-AuNP-AgNP hybrid sensing system.

Detection Probe	LOD (ppm)	Detection Time (min)	Reference
AuNPs	320	6	[33]
Alkyne-AuNPs	3.2	N/A	[45]
DNA-functionalized AgNPs	0.32	N/A	[46]
AgNPs	0.0064	30	[44]
Casein peptide- functionalized AgNPs	0.01	20	[29]
MPD-AgNPs	1.28	10	[47]
Thy-AuNPs-AgNPs	0.03 (UV-vis spectrometry) 0.09 (Smartphone)	15	This work

N/A = not available.

**Table 2:** Application of the Thy-AuNP-AgNP hybrid sensing probe for Cu^2+^ analysis of real water samples on a UV-Vis spectrophotometer and a smartphone-based platform.

Samples	Final Conc. (ppm)	UV-Vis Spectrophotometer			RGB Application		
		Detection Value (ppm)	RSD (%)	Recovery (%)	Detection Value (ppm)	RSD (%)	Recovery (%)
1	0	ND	ND	ND	ND	ND	ND
2	0.2	0.140	3.21	70.14	0.203	9.49	101.74
3	0.4	0.336	17.63	84.01	0.361	8.71	90.32
4	1.0	0.835	14.51	83.60	1.036	6.26	103.59

ND = Not detected.

## Data Availability

Not applicable.
